# UVC inactivation of pathogenic samples suitable for cryo-EM analysis

**DOI:** 10.1038/s42003-021-02962-w

**Published:** 2022-01-11

**Authors:** Jamie S. Depelteau, Ludovic Renault, Nynke Althof, C. Keith Cassidy, Luiza M. Mendonça, Grant J. Jensen, Guenter P. Resch, Ariane Briegel

**Affiliations:** 1 Department of Microbial Sciences, Institute of Biology, Leiden University, Sylviusweg 72, 2333 BE Leiden, The Netherlands; 2 Netherlands Centre for Electron Nanoscopy (NeCEN), Leiden University, Leiden, The Netherlands; 3 Department of Biochemistry, University of Oxford, Oxford, UK; 4 Biology and Bioengineering Department, California Institute of Technology, Pasadena, CA USA; 5 Biology and Bioengineering Department, California Institute of Technology, Pasadena, CA, USA and Department of Chemistry and Biochemistry, Brigham Young University, Provo, UT USA; 6 Nexperion e.U.–Solutions for Electron Microscopy, Vienna, Austria; 7 Present Address: Division of Structural Biology, Wellcome Trust Centre for Human Genetics, University of Oxford, Oxford, UK

**Keywords:** Cryoelectron microscopy, Microbiology

## Abstract

Cryo-electron microscopy has become an essential tool to understand structure and function of biological samples. Especially for pathogens, such as disease-causing bacteria and viruses, insights gained by cryo-EM can aid in developing cures. However, due to the biosafety restrictions of pathogens, samples are often treated by chemical fixation to render the pathogen inert, affecting the ultrastructure of the sample. Alternatively, researchers use in vitro or ex vivo models, which are non-pathogenic but lack the complexity of the pathogen of interest. Here we show that ultraviolet-C (UVC) radiation applied at cryogenic temperatures can be used to eliminate or dramatically reduce the infectivity of *Vibrio cholerae* and the bacterial virus, the ICP1 bacteriophage. We show no discernable structural impact of this treatment of either sample using two cryo-EM methods: cryo-electron tomography followed by sub-tomogram averaging, and single particle analysis (SPA). Additionally, we applied the UVC irradiation to the protein apoferritin (ApoF), which is a widely used test sample for high-resolution SPA studies. The UVC-treated ApoF sample resulted in a 2.1 Å structure indistinguishable from an untreated published map. This research demonstrates that UVC treatment is an effective and inexpensive addition to the cryo-EM sample preparation toolbox.

## Abstract (web summary)

Depelteau et al. present a new method to inactivate cryo-EM samples from pathogenic organisms before imaging using ultraviolet-C radiation in cryogenic conditions. This method allows for the inexpensive preparation of cryo-EM samples with no discernable structural impact of the treatment.

## Introduction

Cryogenic electron microscopy (cryo-EM) has emerged as a powerful technique for determining the structural characteristics of individual proteins, protein complexes, whole viruses, and even intact cells. The major advantage of this method is that it does not require any of the potentially artifact-inducing preparation steps needed for traditional transmission electron microscopy, such as dehydration, staining, or plastic embedding. Instead, the samples are simply flash-frozen into a glass-like ice (vitrified) and remain in a near-native state. These samples can then be directly imaged using cryo-EM, providing high-resolution structural information which in turn enables scientists to answer a wide range of specific biological questions.

The power of this technique is becoming increasingly apparent. For example, cryo-EM has given important insight into how components of SARS-CoV-2, the coronavirus causing the COVID-19 epidemic, interact with host cells, potential drugs, and candidate vaccines^1–5^. However, many of the structure-related studies resulted from either in vitro expressed proteins, isolated proteins from inactive virus, or a virus from the same family but lacks pathogenicity for humans. In other words, the information, while important to the understanding of how this virus infects its host and how to treat it, lacks structural information from the actual pathogen in a natural setting.

This is not only a limitation for the virus, but it is also the case for pathogens in general: the sample preparation for cryo-EM by flash-freezing is optimized to minimize structural damage. Therefore, precautions according to the specific biosafety level of any given sample need to be maintained throughout the entire sample preparation and imaging workflow. This limits the applicability of cryo-EM for obtaining structural information from such infectious pathogens, as cryo-EM facilities may not be equipped or approved to work with pathogens of biosafety level 2 or higher, or researchers are limited to specialized centers which host specialized, dedicated equipment such as a Titan Krios within a BSL2 or higher environment. This is a limiting factor for the research community’s ability to study these medically important organisms in real-life scenarios.

A recent study by Jin et al. demonstrated that ultraviolet (UV) irradiation of cryopreserved mouse embryonic fibroblasts has little-to-no effect on elemental distribution of the frozen hydrated samples^6^. With a similar idea in mind, we began testing whether UV irradiation could be applied to pathogens without compromising the ultrastructure of the samples. We tested this by applying ultraviolet-C (UVC) irradiation to inactivate two pathogenic organisms. UVC irradiation was chosen because of its well-described ability to inactivate pathogens^7–9^. Inactivation occurs by altering the DNA of the organism, leading to the prevention of transcription and replication^10,11^. UVC irradiation is also known to impact proteins, mainly by disulfide bond breakage and the creation of reactive oxygen species^12,13^. A combination of the above effects leads to the loss of viability of the organism, but the detailed effects on the structure of the pathogen are not well documented.

Here we describe a simple, inexpensive proof-of-principle prototype for administering UVC irradiation to cryo-EM samples under cryogenic conditions. We initially tested this device on two model organisms: the pathogenic bacterium *Vibrio cholerae* and the bacteriophage ICP1, a virus-like particle that infects *V. cholerae*. These organisms were chosen to test the applicability of the UVC inactivation for two different cryo-EM methods. The bacterium was used for cryo-electron tomography (cryo-ET) and sub-tomogram averaging (STA) workflows. These methods are used to study heterogeneous samples like whole bacterial cells and typically results in resolutions limited to macromolecular resolution (2–4 nm, 20–40 Å). Single particle analysis (SPA) was used to evaluate the ICP1 bacteriophage sample, a method which allows the structure determination of identical particles to higher resolution (below 1 nm, 10 Å). Finally, we used SPA of the protein apoferritin (ApoF) to determine structural changes at high (near-atomic) resolution that might result from UVC irradiation. ApoF is a common validation standard used in the EM community and resolutions of better than 3 Å are routinely achieved.

In this study, we show that UVC irradiation effectively inactivated both the pathogenic bacterium and the bacteriophage. We further show that the effects of this treatment on the structural information are non-discernable in the tested STA and SPA samples. Our study demonstrates that UVC irradiation of vitrified pathogen samples is a promising alternative to chemical fixation or the use of in vitro systems when access to a cryo-EM facility with sufficient biosafety clearance is not feasible. Combined with a simplified plunging device, it may be possible to freeze and UVC-treat samples locally, which can then be imaged at a lower safety level without compromising the structural information. In summary, this study provides compelling evidence that UVC irradiation of vitrified samples may be a viable solution for structural investigations of a broad range of pathogen with cryo-EM.

## Results

### UV inactivation of *Vibrio cholerae*

As an initial test of the UV inactivation device, we determined the UVC exposure time necessary to inactivate the pathogen, *V. cholerae A1552 WT*. Our experiments showed a reduction in vitrified, viable cells after 20 s of UVC exposure, and complete inactivation with 30 s of UVC exposure (Fig. S2). Based on this information, we chose 30 s as the timepoint for inactivation and subsequent imaging.

**Fig. 1:**
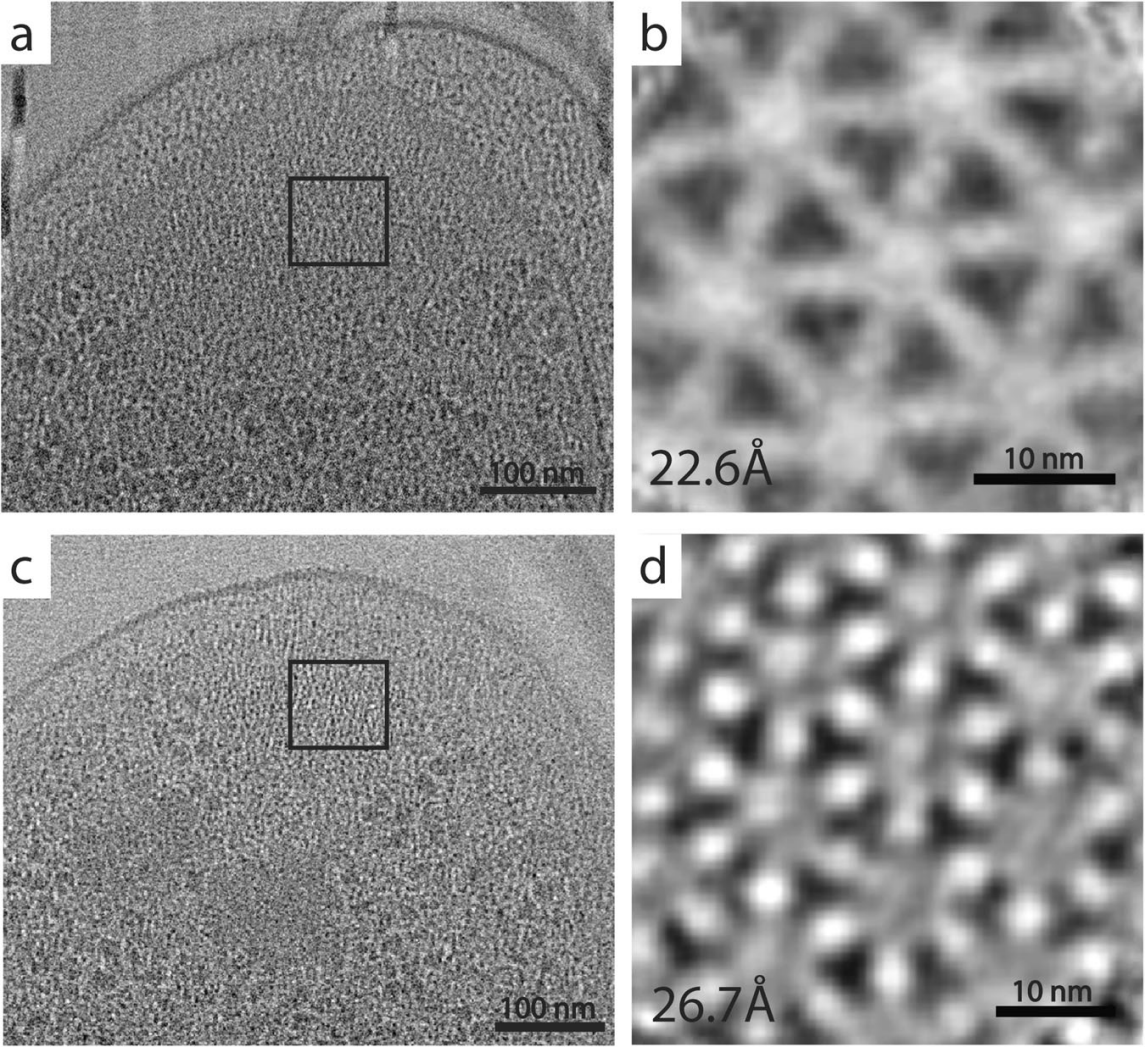
Effect of UVC irradiation on *Vibrio cholerae and its chemotaxis array*. Representative tomographic slices showing the flagellar pole region of untreated (**a**) and UVC-treated (**c**) *V. cholerae* cells, highlighting the top view of the F6 chemotaxis array (black box). **b**, **d** Results of sub-tomogram averaging of F6 chemotaxis cluster, showing the typical hexagonal arrangement of the trimer-of-dimer receptors in extended arrays.

Following the UVC treatment of the *V. cholerae* cryo-EM sample, the grids were transferred to a Titan Krios electron microscope for data collection. To determine the effect of the UVC irradiation, we collected tilt series of the flagellar poles of individual cells, with the aim of imaging the F6 chemotaxis array, which are located at the same pole. Chemotaxis arrays are well-studied by ECT and STA, and thus provided a good model system for determining of macro-level damage to cells. Following data collection and subsequent processing, tomograms with top views of the F6 chemotaxis array were identified and further processed using STA (Fig. 1a, c). For the control sample, and using 219 particles, we were able to achieve a resolution of 22.6 Å (Fig. 1b), whereas with the UVC-treated sample and 170 particles, we were able to achieve a final resolution of 26.7 Å (Fig. 1d).

### UV inactivation of ICP1 phage

**Fig. 2:**
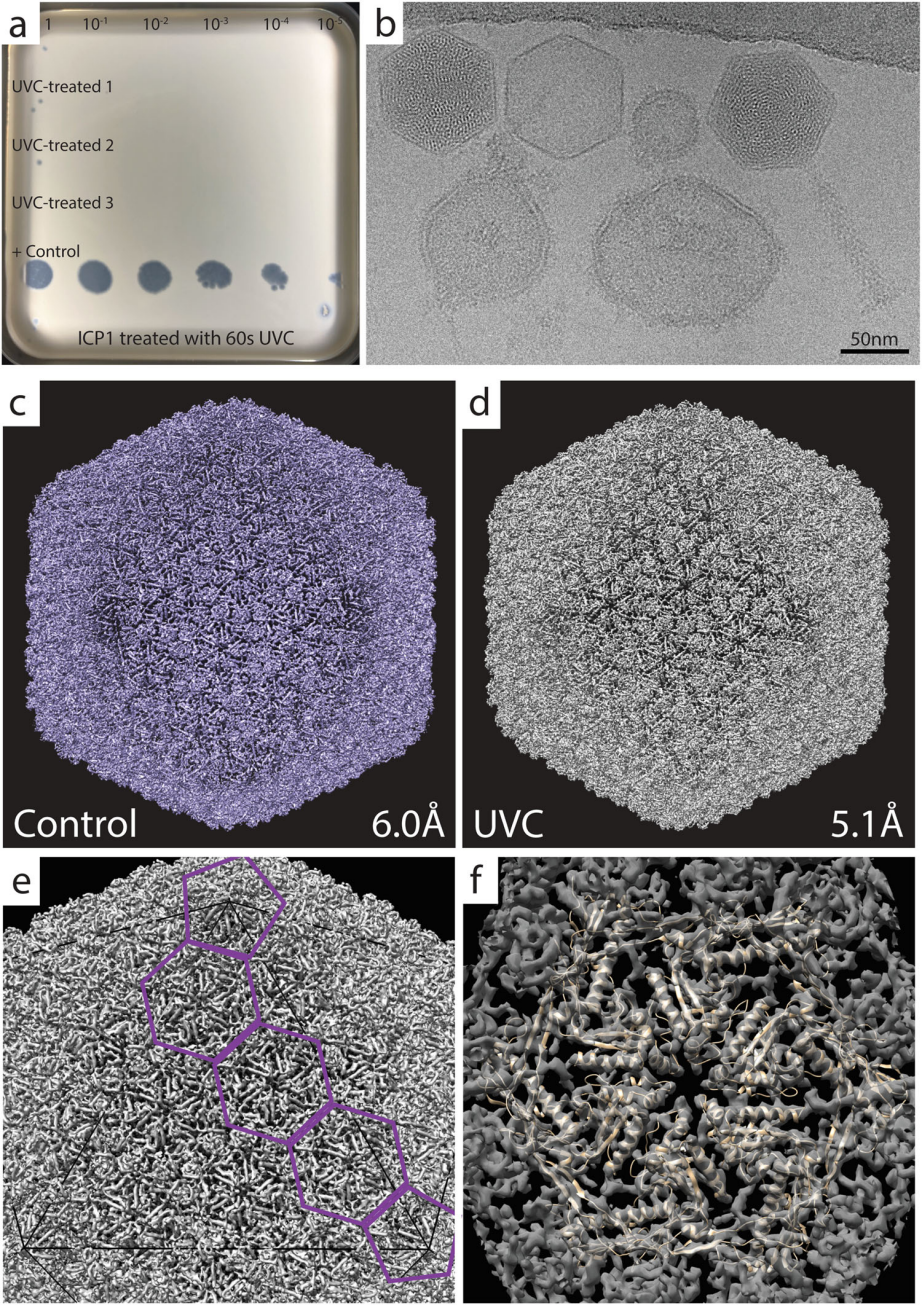
UVC treatment and SPA of ICP1. **a** UVC treatment of ICP1 resulted in a reduction in viable phage. Rows 1–3 are UV treated and show 0–2 infective phages in three replicates when undiluted (dilution factor noted at the top of panel). Row 4 is a negative control, a grid containing ICP1 that remained in the grid box during treatment. **b** A representative micrograph of UVC-treated ICP1 showing an assembled phage (far right), an empty and full head (middle/left), and liposomes (round, bottom). **c**, **d** SPA analysis resulted in structures of the control and UVC-treated ICP1 phage with a resolutions of 6.0 Å and 5.1 Å, respectively. **e** Determination of the T number = 13, which a measure of the icosahedral symmetry. **f** Flexible fitting of putative major capsid protein, gp122, as a hexamer, and docked into the ICP1 capsid of the UVC-treated structure noted in **d**.

The ICP1 bacteriophage was chosen as a model for a virus-like particle. Initially, we used the same protocol for UVC treatment as we had applied to the bacterial sample. However, this protocol did not yield consistent reduction in active ICP1. We postulated that a two-sided irradiation step might be necessary due to the size of ICP1 (virus particle of 90 × 210 nm compared to an average *V. cholerae* cell of 500 × 1500 nm). We speculate that the small bacteriophage may gain access to both sides of the grid, for example due to a broken carbon layer. This in turn could protect the ICP1 phage due to shadowing caused by the grid bars. Therefore, we repeated the protocol with the additional irradiation step on the backside of the grid. This modified protocol indeed allowed us to achieve a consistent reduction of at least 99.99%. The two-sided UVC treatment required 60 s for the carbon side combined with 30 s for the copper side (Fig. 2a). We then UVC irradiated a new set of ICP1-containing grids, confirmed inactivation of phage, and used the remaining grids for imaging (Fig. 2b).

Following imaging, the number of particles and classes was determined before subsequent processing. For the UVC-treated sample, 6577 particles were separated into two classes: capsid containing DNA and empty capsids. Only capsids containing DNA were used for 3D classification and refinement. Ultimately, 2845 particles resulted in a final resolution of 5.1 Å for the UVC-treated capsid and a final resolution of 6.0 Å using 4096 untreated particles (Fig. 2c, d). A comparison of the two structures using Chimera:Fit in Map yielded a correlation value of 0.9815. The achieved resolutions allowed for the identification of the T number (T = 13; Fig. 2e^14^); which is a metric for the icosahedral symmetry, as well as the docking of a model of the putative major capsid protein (gp122) that was obtained using AlphaFold (Fig. 2f;^15^).

To compare the local quality of the two maps, we used each to structurally refine a hexameric assembly of major capsid proteins (Fig. 2f). For this purpose, we carried out molecular dynamics flexible fitting (MDFF) simulations^16^, which use a density-derived potential to optimize model-map overlap. The refined hexamer models were nearly identical, possessing a backbone root-mean-square-deviation (RMSD) of 1.9 Å for all residues and 1.1 Å for non-loop residues. The backbone RMSD between the refined and initial hexamer models was 3.4 and 3.3 Å, respectively, for the untreated and UVC-treated maps. The structural information contained in both maps is therefore of comparable quality.

### UV inactivation of ApoF

Finally, we wanted to determine the effect of UVC irradiation at resolutions similar to X-ray crystallography, which allow the visualization of individual amino acids. To do this, we used the protein complex, apoferritin (ApoF). ApoF is commonly used in EM facilities to determine the performance of microscopes and it has been well characterized by the scientific community using cryo-EM and x-ray diffraction. Using the same UVC exposure time as ICP1, grids containing apoferritin were treated with UVC for 60 s on the carbon side and 30 s on the copper side. These grids were subsequently imaged and the data processed using SPA (Figs. S3 and S4). Our experiments demonstrate that we were able to achieve a final resolution of 2.1 Å in the UVC-treated sample, which is on par with structures published in the Protein Data Bank (https://www.ebi.ac.uk/pdbe/node/1).

**Fig. 3:**
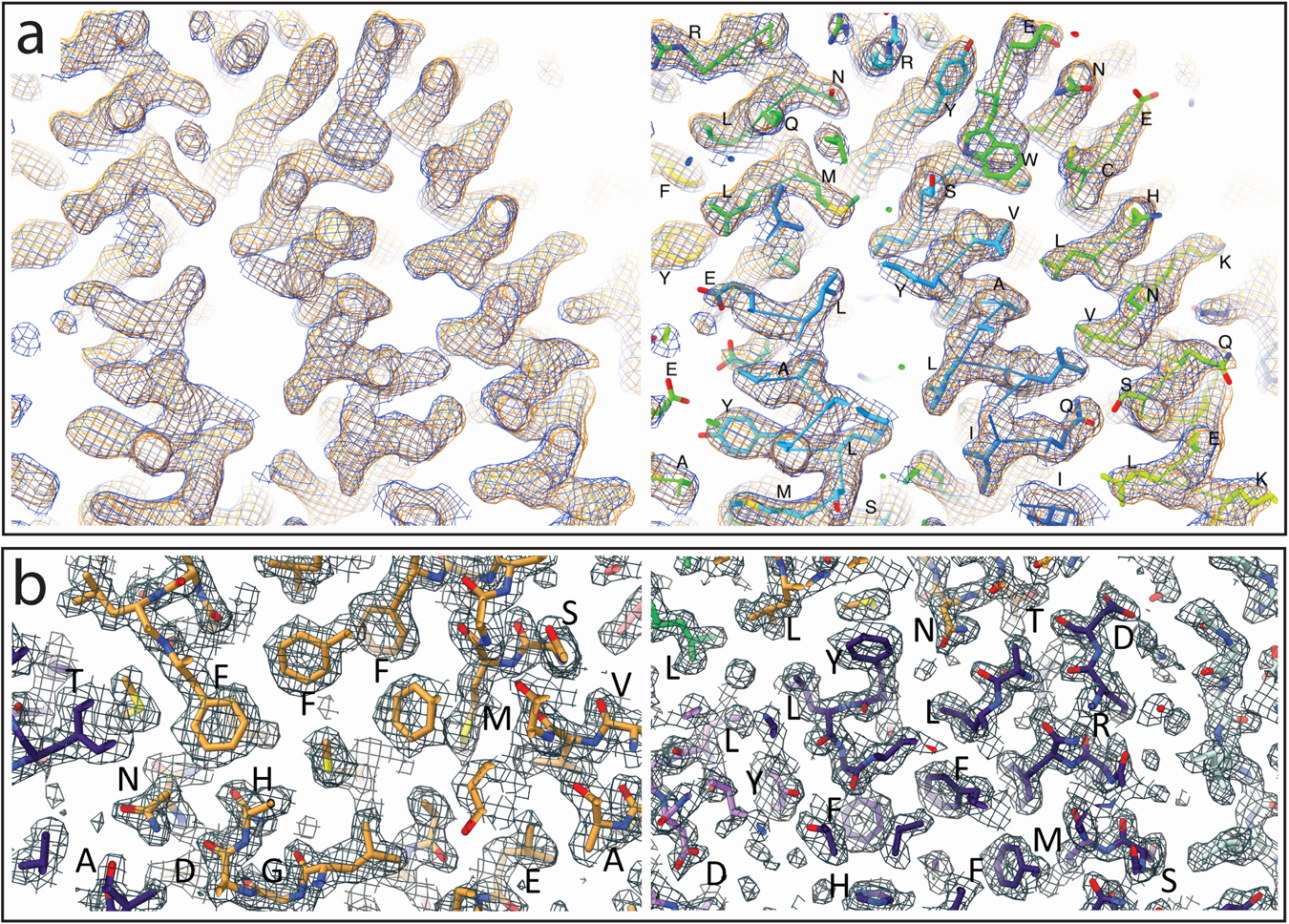
Representative ApoF amino acids show no difference between the UVC-treated ApoF cryo-EM map and the crystal structure. **a** A comparison of the UVC-treated ApoF map (blue) with a previously published, untreated sample map (orange; EMD-3853) (left). The same map UVC-treated ApoF map superimposed with the fitted model (PDB-5n27) confirms no differences (right). **b** Close-up view of UVC-treated ApoF map with same fitted model as in **a**, highlighting no difference between representative amino acids that may be impacted by UVC irradiation.

A comparison of the map resulting from a UVC-treated apoferritin sample with untreated, published maps found no difference between the two structures (Fig. 3a; EMD-3853, PDB5N27^17^). A closer analysis of selected regions of the structure of the treated apoferritin sample highlight the structural preservation (Fig. 3b).

## Discussion

UVC irradiation is a widespread technique used for disinfection^18,19^. Specifically, it has been used for inactivation of viruses^9^. In this paper, we demonstrate that UVC irradiation is also suitable for inactivating pathogens in cryogenic samples intended for structural analysis by cryo-EM. Our results confirm a reduction and or elimination of the ICP1 bacteriophage and its target organism, *V. cholerae*. We characterized the effects of this treatment by comparing cryo-EM data of control (untreated samples or previously published structures) with samples after inactivation. Here, we show at multiple scales that UVC irradiation has no discernable effect on the structural information of the biological cryo-EM samples.

Cryo-EM has become a valuable method for understanding bacterial cell biology. Using cryo-ET, the organization of bacterial cells and their molecular machines have become accessible for structural analysis. Thus, we began by determining the effects of UVC irradiation on a widely studied bacterium. We show that while the bacterial pathogen *V. cholerae* is completely inactivated by UVC, the cell’s ultrastructure is indistinguishable from untreated samples. More specifically, using the F6 chemotaxis array as an example, we show that the STA results (reaching resolutions between 2 and 4 nm) are similar to the published literature for complexes of similar size and in cells with similar thicknesses^4,20–22^.

We next wanted to test the suitability of this treatment method on a virus-like particle. We therefore tested the UVC treatment on ICP1, a bacteriophage that infects *V. cholerae*. Similar to the bacterial sample, the bacteriophage was also effectively inactivated using the UVC treatment of the cryogenic samples. The resolution reached here (~5–6 Å) allowed for the identification of individual alpha helices, turns within the protein, and groups of beta sheets. This information was used to gain structural information about the ICP1 capsid, including its overall structure as demonstrated by its T number, the spike protein complex at the vertices, and the docking of the putative major capsid protein into the capsid hexamer. This result further illustrated the suitability of the UVC inactivation method for low-resolution SPA of virus-like particles.

Finally, we demonstrated the applicability of this method for high-resolution SPA. At resolutions of better than 3 Å, structural damage would likely be visible by the lack of disulfide bonds and damage to aromatic amino acids. Our experiments show that UVC irradiation had no impact on our ability to obtain a final structure of ApoF at a resolution of 2.1 Å (Figs. 3 and S4). This resolution is comparable to other reported structures (EMD-3853, PDB5N27^17^). A closer examination of the treated and untreated data did not reveal any noticeable differences between the two structures (Fig. 3). While there are no noticeable structural differences in the samples used in this study, it is important to note that undetected damage could be possible, and that this proof-of-concept study does not guarantee that all proteins will behave similarly.

The results of this study are of particular significance to groups studying pathogens with a potential for human infection. We show a 5-fold reduction in infectious particles when ICP1 is treated with UVC. The treatment, combined with the reduction of infectious particles that are associated with the blotting step of plunge freezing reduce the remaining viable particles. Furthermore, in the case of unintentional dropping of the sample at any point during loading, imaging or removal from the cryo-EM, the particles are subject to the ambient environment or the vacuum of the microscope, which further increases the likelihood of their inactivation. Therefore, pathogenic specimen would likely be safe to image on a cryogenic electron microscope approved for lower safety levels.

This research did not come without some challenges. For instance, our initial experiments to determine PFU used the same strain of *V. cholerae* as used for the cryo-ET and STA. However, we found that this strain and antibiotic combination gave inconsistent PFU counts and thus necessitated a switch to a strain more similar to the ICP propagation protocol. When working with the ICP1 sample, we also found many factors could influence the reduction in infectious particles, including contamination from forceps, bending of the grids during transfer (which can result in breaking of the carbon layer providing opportunities for the sample to attach to both sides of the grid), and the presence of outer membrane vesicles/lipid containing particles in the sample preparations. Thus, it will be important to determine the needs for their specific sample prior to imaging.

Together, this research demonstrates that UVC irradiation of vitrified samples can provide important structural information for a variety of samples and across scales. We believe this information is especially useful for labs who study pathogens that may not be approved for imaging at their local, regional, or national facilities. We demonstrate that UVC treatment of the samples directly on the grid preserves the structural information while rendering the pathogen with reduced or no infectivity. It is important to note that the inactivation protocol will have to be adapted to each pathogen of interest. Researchers will need to work with their local biosafety officers to determine the applicability to their specific pathogen and research environment. Regardless, this inactivation method provides an affordable, straightforward method to inactivate pathogens for cryo-EM studies and will be useful for all laboratories lacking access to cryo-EM facilities that are certified for higher biosafety level microorganisms.

## Methods

### Bacteria and bacteriophage strains and conditions

*Vibrio cholerae* strains A1552 wildtype (WT; rifampicin resistance, 100 µg/ml), N16961 WT (streptomycin resistance, 100 µg/ml), and N16961-TndsRed (gentamycin resistance, 10 µg/ml) were provided by Dr. Melanie Blokesch (Ecole Polytechnique Fédérale de Lausanne, Switzerland). *V. cholerae* were grown overnight in lysogeny broth (LB) containing antibiotic at 30 °C with shaking at 180 rpm. Equal volumes of an overnight culture and sterile 50% glycerol or 10% DMSO were mixed, frozen in liquid nitrogen, and stored at −80 °C until use.

The ICP1 bacteriophage was provided by Andrew Camilli (Tufts University, Massachusetts, USA). The ICP1 phage was propagated using *V. cholerae* N16961 WT as described previously^23^. Briefly, 10^6^ ICP1 phage was added to a 50 ml culture (starting OD_600_ = 0.2) containing 5 µM CaCl_2_. The culture was incubated for 4–6 h at 30 °C with shaking at 180 rpm and the bacteria was pelleted by centrifugation (5000 *g* at 4 °C for 30 m) and discarded. The supernatant is filtered through a 0.20 µm filter, subsequently ICP1 is precipitated out using 20% PEG8000/2.5 M NaCl solution overnight. Finally, ICP1 is pelleted by centrifugation (3000 *g* at 4 °C for 1 h) and dissolved in phage storage buffer (100 mM NaCl, 10 mM MgSO_4_, 10 mM Tris-HCl (pH 7.5), 1 mM EDTA) and stored at 4 °C until use.

### Sample preparation

*V. cholerae* was streaked onto a selective LB plate and grown overnight at 30 °C and stored at room temperature. The night before the freezing session, several colonies were resuspended in liquid LB containing antibiotic and grown overnight at 30 °C with shaking at 180 rpm. An aliquot of this culture was used for freezing as described below. For ICP1, an aliquot from the phage stock was used directly for sample freezing. To minimize lipid vesicles in the ICP1 sample, Tween-80 (Sigma Aldrich, St. Louis, MO, USA) was added to the UVC-treated sample shortly before freezing to a final concentration of 0.05%.

The bacterial and phage samples were prepared using a Leica EM GP (Leica Microsystems, Wetzlar, Germany). For the bacterial sample, 15 nm gold beads was added to the bacteria prior to freezing (Cell Microscopy Core, Utrecht University, Utrecht, The Netherlands). For both samples, 3 µl was applied to a glow discharged Quantifoil R2/2, 200 mesh Cu grid (Quantifoil Micro Tools GmbH, Jena, Germany), and incubated prior to blotting (30 s for *Vibrio cholerae* or 10 s for ICP1) at 18 °C with ~90% relative humidity. The grid was blotted for 1 s and automatically plunged into liquid ethane. Vitrified samples were transferred to storage boxes and stored in liquid nitrogen until use. All grids were screened using a Talos L120C cryo-electron microscope (Thermo Fisher Scientific (TFS), Waltham, MA, USA) equipped with a 626 side entry holder (Gatan, Inc., Pleasanton, CA, USA) to determine suitability for data collection.

Human apoferritin (ApoF) sample was used at a concentration of 4 mg/mL and applied to glow discharged Quantifoil R2/2, 200 mesh grid before being double-side blotted for 3 s in a Vitrobot Mark IV (TFS) and plunge frozen into liquid ethane. Frozen samples were stored in liquid nitrogen until UVC treatment and imaging.

### UV irradiation device and protocol

The UV irradiation device was constructed using easy-to-acquire materials. Working with our local fine mechanical department, we created a sandwich of three quartz glass slides (Fig. S1a). Quartz glass was chosen because it does not interfere with UVC radiation (Alfa Aesar (TFS), Kendal, Germany; noted by manufacturer as offering optical transmission of UV light). This sandwich design allows for the accurate placement of the frozen grids during the UVC irradiation process (Fig. S1b). A holder for the grid box and quartz slide sandwich was designed using styrofoam, and inserted tightly into a modified blue foam liquid nitrogen dewar (Fig. S1c; Spearlabs Cryogenic Products). The styrofoam holder contains a shallow well sized to the assembled quartz glass sandwich, and is lined with aluminum foil to encourage omnidirectional irradiation (Fig. S1c arrow). This assembly allowed for the loading and unloading of grids under liquid nitrogen conditions (Fig. S1d), and it permitted the placement of the UVC light source directly above the samples.

The UVC light source (6 W Germicidal Light T5 Tube UVC Sterilizer, Lcamaw via Aliexpress.nl) was mounted to the underside of a box to allow the placement of the blue sample assembly and a UVC sensor (UV Light Meter LS126C, Linshang Technologies, China) directly below the light source (Fig. S1e). The UVC sensor was placed adjacent to the sample assembly at sample height to confirm irradiation and dose rate. At the sample, the dose rate from the light source reached a maximum of 490 µW/cm^2^, as determined with the final reading before discontinuing UVC treatment. Based on this setup, a box was designed to properly contain the light source, sensor, and sample assembly (Fig. S1f).

Samples were irradiated as follows: During each irradiation experiment, four sample-containing grids were processed. Three of the grids are transferred to a position in the quartz grid holder. The fourth grid was left in the grid box with the lid closed to act as a control (grid box lid is opaque and grid remains perpendicular to the light source, preventing the passage of UVC to the sample). The liquid nitrogen level was then raised to a level of 0.5 cm above the sample, which we have marked on inside of the blue foam container. Prior to irradiation, the UVC light was switched on for 1 min (prewarming step) and then switched off. Immediately after sample assembly and pre-warming, the lamp is placed over sample assembly, and the lamp switched on for the treatment time. For the bacterial sample, grids were treated on the carbon side only; for the bacteriophage sample, irradiation occurred on both the carbon and copper side of the grids (by inverting the sandwich in the liquid nitrogen/vapor layer). Once treatment was complete, the grids are transferred back to the grid box and stored in liquid nitrogen until the confirmation of inactivation and or imaging.

### CFU and PFU

To determine the exposure time for UVC treatment of *V. cholerae* or the ICP1 bacteriophage, the viability of the samples was determined by either colony forming units (CFU) for the *V. cholerae* WT, or plaque forming units (PFU) for the bacteriophage exposed to *V. cholerae* N16961-TndsRed. The treated grids were transferred to LB, pulse vortexed several times to resuspend the bacteria/phage and then subjected to serial dilution. CFUs were determined by plating 100 µl of the serial dilution onto non-selective LB plates and incubated overnight at 30 °C. The PFU were determined as follows: *V. cholerae* N16961-TndsRed containing soft agar was overlayed on square LB plates containing 10 µg/ml gentamycin. 5 µl from each dilution step was spotted onto the top layer and the plate left to grow overnight at room temperature. Clearance zones were noted the following day, and PFU were roughly determined. The duration of UVC exposure to inactivate greater than 99.99% of the phage was determined based on the amount of time needed to prevent ICP1 predation of *V. cholerae*, as demonstrated by the lack of clearance zone.

### Statistics and reproducibility

Experiments to confirm UVC inactivation of *V. cholerae* and ICP1 were carried out in triplicate and inactivation was confirmed by CFU and PFU, respectively. For the *V. cholerae*, the average and standard error was determined for each timepoint and plotted on a logarithmic scale. The data associated with the graph are presented as an insert into the graph sample (Fig. S2). Subsequently, a separate set of vitrified samples were treated with UVC for a length of time resulting inactivation, one grid was used to confirm inactivation, the remaining grids were used for data collection by cryo-EM. For the SPA of ApoF, the sample was treated with UVC for the same time as the ICP1 sample, and one grid was imaged for data collection.

### Imaging conditions

#### Cryogenic electron tomography

Suitable grids containing vitrified, UVC-treated *V. cholerae* were clipped and loaded into a CS-corrected Titan Krios (TFS) equipped with a K2 direct electron detector and a post-column energy filter (Gatan, Inc.) set to zero loss imaging with a 20 eV slit. Targets were chosen based on the presence of the flagellar pole in a hole. This extracellular feature is a good indicator for the presence of the F6 chemotaxis array that is located at the same cell pole. A tilt series of each target was collected using SerialEM set to a dose symmetric tilt scheme between −54° and 54°, with 2° increments, and a pixel size of 3.49 Å^24,25^. A defocus of −8 µm and a cumulative dose of 140 e−/Å were used as targets.

For the untreated data set, we used data collected during a previous session. This data was collected on a Titan Krios (TFS) microscope equipped with a K3 BioQuantum direct electron detector and energy filter (Gatan, Inc) set to zero loss imaging with a 20 eV slit. Whole cells in a hole were selected as targets. Tilt series were collected using a bidirectional scheme between −60° and 60° with 2° increments, and a pixel size of 5.86 Å. The target defocus was set to −8 µm and the estimated total dose 170 e−/Å.

#### Single particle analysis

Suitable UVC treated and untreated ICP1-containing grids were clipped and loaded into a Titan Krios (TFS) microscope equipped with a K3 BioQuantum direct electron detector and energy filter (Gatan, Inc.) set to 20 eV. Micrographs were collected using the TFS EPU software equipped with AFIS (aberration free image shift) with a pixel size of 0.685 Å in super-resolution mode. Defocus was set cycled between −1 and −3 µm, and a dose of 34 e/Å^2^ per image.

Apoferritin samples were treated as described in Diebolder et al.^26^. Briefly, grids were loaded into a Titan Krios electron microscope (TFS) operated at 300 kV, equipped with a Gatan K3 BioQuantum direct electron detector (Gatan, Inc). Movies with 50 frames and an accumulated dose of 50 electrons/Å2 were acquired in super-resolution counting mode using EPU (TFS) at the magnification of ×130,000, corresponding to a calibrated pixel size of 0.328 Å/pixel with a defocus range of −0.5 to −2.5 µm. A total of 1040 movies were collected at The Netherlands Centre for Electron Nanoscopy (NeCEN). Detailed data acquisition parameters are summarized in Table 1.

**Table 1:** Cryo-EM data collection, refinement and validation statistics.

	hApoF (EMDB-13364) (PDB 7PF1)	UVC-treated ICP1 (EMDB-13402)	Wildtype ICP1 (EMDB-13403)
Data collection and processing			
Magnification	130,000	64,000	64,000
Voltage (kV)	300	300	300
Electron exposure (e−/Å^2^)	50	34	34
Defocus range (μm)	−0.5 to −2.5	−1 to −3	−1 to −3
Pixel size (Å)	0.328	0.685	0.685
Symmetry imposed	O	I	I
Initial particle images (no.)	251,350	72,048	25,285
Final particle images (no.)	26,735	2845	4096
Map resolution (Å)	2.1	5.1	6.0
FSC threshold	0.143	0.143	0.143
Map resolution range (Å)	2.0–2.5		
Refinement			
Initial model used (PDB code)	5N27		
Model resolution (Å)	2.1		
FSC threshold	0.143		
Model resolution range (Å)	2.0–2.5		
Map sharpening *B* factor (Å^2^)	−44		
Model composition			
Non-hydrogen atoms	41,646		
Protein residues	4350		
Ligands	N/A		
*B* factors (Å^2^)			
Protein	15		
Ligand	N/A		
R.m.s. deviations			
Bond lengths (Å)	0.002		
Bond angles (°)	0.434		
Validation			
MolProbity score	1.36		
Clashscore	6.54		
Poor rotamers (%)	0.25		
Ramachandran plot			
Favored (%)	98.28		
Allowed (%)	1.50		
Disallowed (%)	0.22		

### Data processing

#### *Vibrio cholerae*

Motion correction and tomogram generation were done using the IMOD image processing suite^27^. In brief, once the tilt series were motion-corrected using the alignframes function, the batchruntomo function was used to generate the initial tomograms to determine if the structure of interest was present in the cell (final bin = 3 without CTF correction;^28^). Tomograms containing the feature of interest were further processed by refining the bead model, or using patch tracking if insufficient beads were present. Subsequently, the boundary model was improved, CTF correction was applied, and a back-projected, SIRT-like filtered tomogram was generated^29^. A final bin of 2 was used for the UVC-treated data (pixel size = 6.98 Å) and a final bin of 1 for the untreated data (pixel size = 5.86 Å).

The resulting tomograms were used for STA using the Dynamo imaging suite^30,31^. Subtomograms were manually picked from chemotaxis array top views. Two rounds of iteration steps were performed, using a template generated from particles picked from a single tomogram, and a mask that encompassed a single hexagon. All final maps were calculated from weighted back-projection SIRT-like filtered tomograms. The resolution of the final maps were calculated using ResMap^32^.

#### ICP1

Reconstruction of the ICP1 capsid was done using Relion 3.1.2^33^. The data was binned by 4 for the initial particle picking, 2D, and 3D refinements. GPU enabled MotionCorr was used to correct particle movement as part of the Relion processing suite^34^ and gCTF was used for contrast transfer function (CTF) estimation^35^. Initially, 150 phage full and empty heads were manually picked for 2D classification to generate reference templates for autopicking. Following autopicking and extraction, 2D classification was performed using the T4 phage head, PDB 8661, filtered to 70 Å as a reference^36^. 3D classification was performed with a subset of particles using the 2D classification full head as a template. The full head class was auto-refined using “I” symmetry (icosahedral). Post-processing used a mask generated by Relion. Particles were re-extracted with a pixel size of 1.637, 3D autorefine and post-processing was repeated yielding the final structure.

#### Apoferritin

RELION-3.1 beta software was used for ApoF image processing^33,37^. Collected movies were subjected to beam induced drift correction using MotionCor2, the CTF was estimated by CTFFIND-4.1.18^34,38^. RELION Gaussian picker was used to automatically pick 251,350 particles. After 2 rounds of 2D classification, false positive and contaminating features were discarded resulting in a 66,920 particles dataset. Ab-initio model generation followed by 3D classification and 3D refinement yielded a 2.6 Å map. “O” symmetry was applied at the initial model generation and 3D refinement steps. Corresponding particles were subjected to CTF refinement for optical and beam-tilt and aberration correction, as well as per-particle defocus, and per-micrograph astigmatism correction followed by Bayesian polishing^39,40^. A second 3D refinement was then performed yielding a 2.1 Å map.

Map resolution were estimated at the 0.143 criterion of the phase-randomization-corrected FSC curve calculated between two independently refined half-maps multiplied by a soft-edged solvent mask. Final reconstructions were sharpened and locally filtered in RELION post-processing. Model refinement and validation statistics are summarized in Table 1. Maps were displayed using UCSF ChimeraX^41^.

#### Molecular modeling and simulations

A model of the major capsid protein was constructed using the gp122 sequence (YP_004251064.1) and AlphaFold Colab^15,42^. A hexameric complex was then assembled into each map by rigid docking using UCSF Chimera v1.13^43^. A 5-ns MDFF simulation was performed on each hexamer using NAMD v2.13 and the cascade-MDFF protocol with default parameters^16,44^. Fittings were carried out with an MDFF coupling constant of 0.1 and symmetry restraints applied on the backbone atoms of each monomer. Structural visualization and trajectory analysis were performed in VMD v1.9.4^45^.

### Reporting summary

Further information on research design is available in the Nature Research Reporting Summary linked to this article.

## Supplementary information

Supplemental Material

Reporting Summary

## Data Availability

The structures resulting from single particle analysis of the untreated and UVC-treated ICP1 bacteriophage data (EMD-13403, EMD-13402), and the UVC-treated ApoF (EMD-13364; PDB ID: 7PF1) data have been deposited in the EMDB.
