# Supplementary material for: UVC inactivation of pathogenic samples suitable for cryo-EM analysis
Source: Commun Biol. 2022 Jan 11;5:29. doi: 10.1038/s42003-021-02962-w (PMC8752862; doi:10.1038/s42003-021-02962-w)
Supplement: Supplementary file 1 — Supplemental Material [file 42003_2021_2962_MOESM1_ESM.pdf]

UVC inactivation of pathogenic samples suitable for cryo-EM analysis

Supplementary Materials

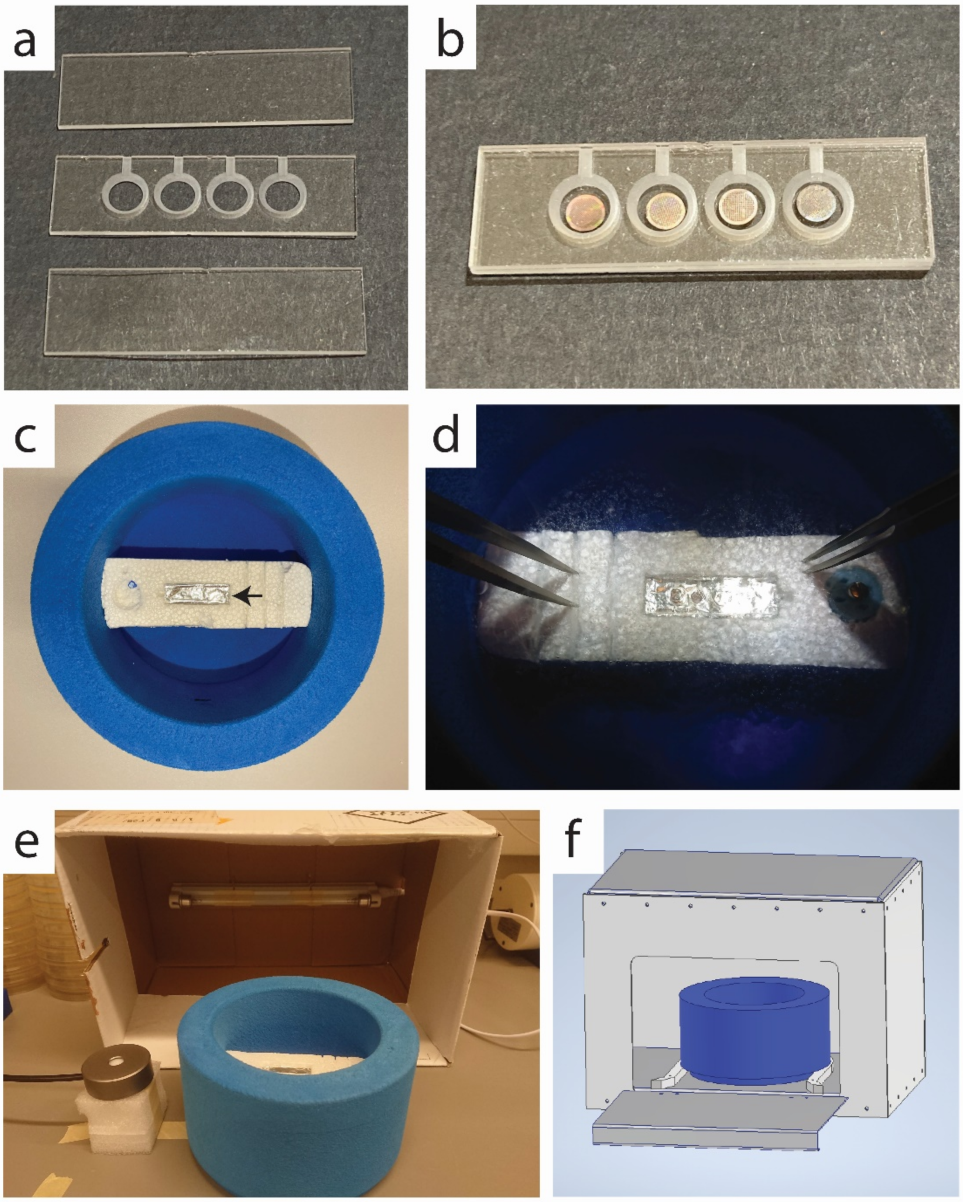

Figure S1. Details of UV inactivation device

Sample containing grids can be loaded into a quartz glass grid holder (unassembled (a) and assembled with grids (b)) under liquid nitrogen conditions. A foam dewar contains a Styrofoam platform for the placement of the quartz glass sample holder during assembly and inactivation, and includes a place for the grid box, an aluminium-lined well for the assemble sample holder (black arrow) and slots for storing each quartz plate (at room temperature (c) and cooled with liquid nitrogen (d)). The initial design of the UV light box mounted the light source to the inside of a cardboard box that could be placed over the cooled sample and UV sensor (e). The final design of the UV light box allows for insertion of the foam dewar into the side (visualized in f).

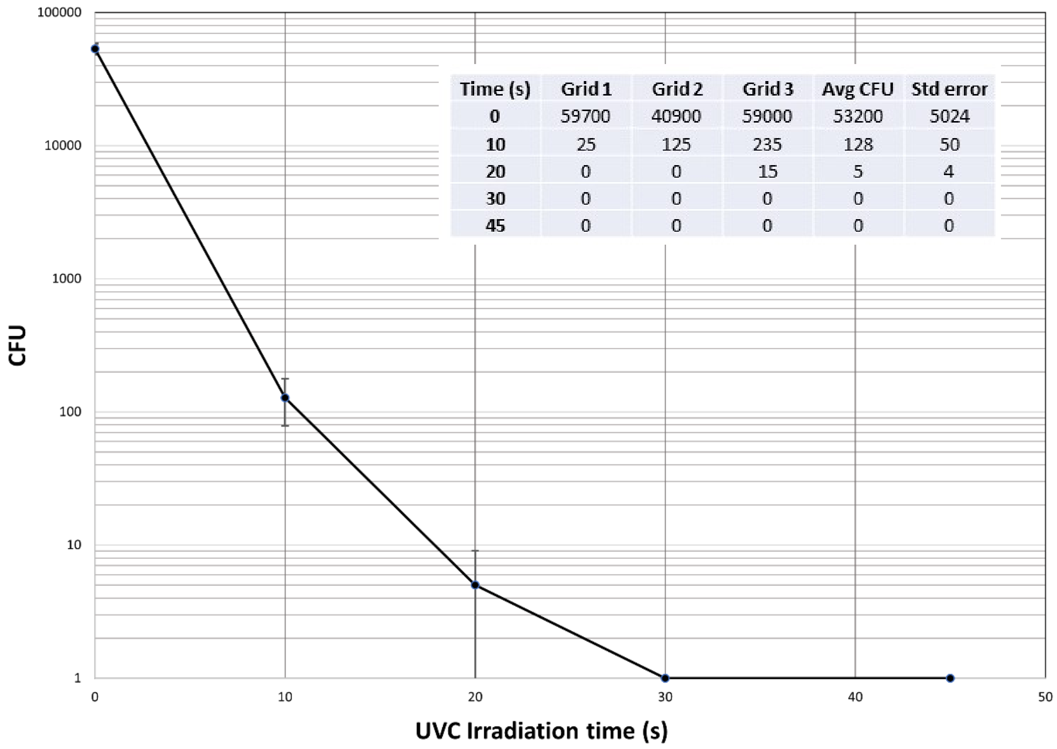

Time (s)	Grid 1	Grid 2	Grid 3	Avg CFU	Std error
**0**	59700	40900	59000	53200	5024
**10**	25	125	235	128	50
**20**	0	0	15	5	4
**30**	0	0	0	0	0
**45**	0	0	0	0	0

Figure S2. Inactivation of *Vibrio cholerae* by UVC.

Three grids for each time point were treated with UVC and subsequently serially diluted to determine colony forming units (CFU). After 30 s of UVC treatment, the viability of *V. cholerae* was reduced by 100%. The average and standard error was determined for each timepoint and plotted on a logarithmic scale. The data associated with the graph are presented as an insert into the graph.

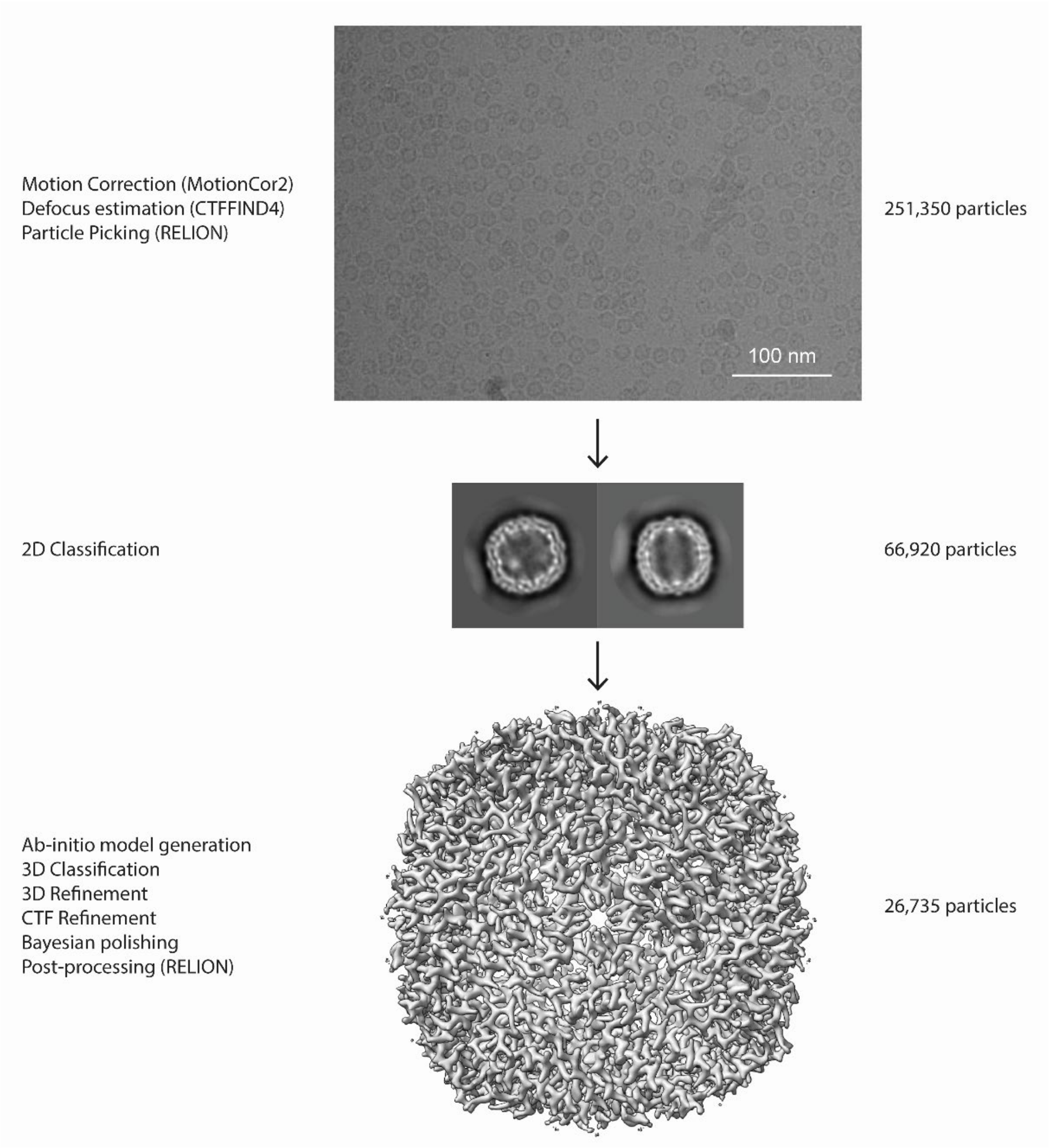

Figure S3. Cryo-EM data processing workflow

A typical micrograph shown as well as representatives 2D classes. 3D classification was performed to distinguish heterogeneity in the sample and the final refined map is shown.

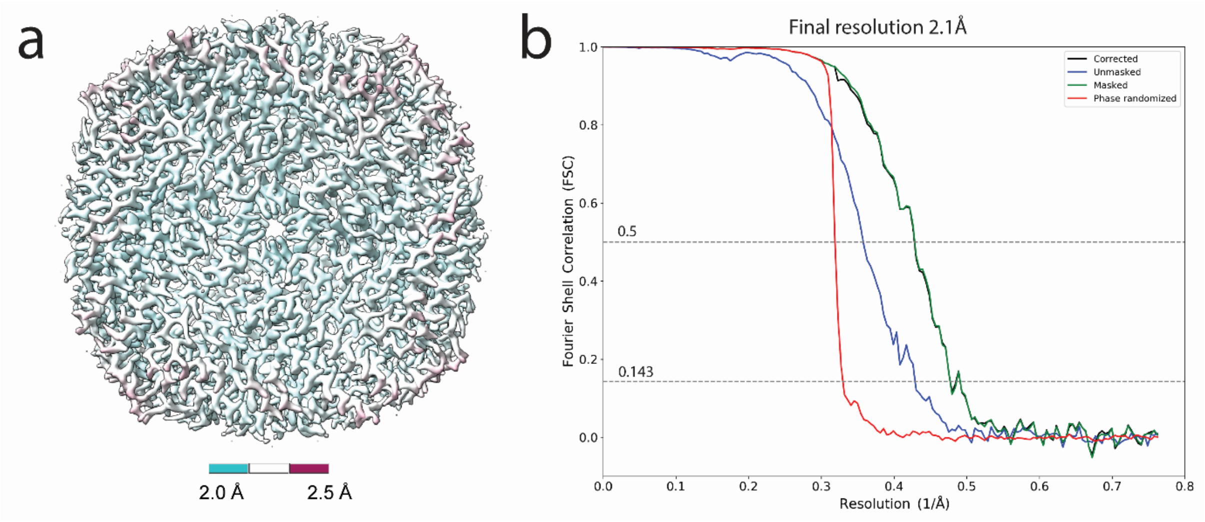

Figure S4. Resolution assessment of cryo-EM structure

a. Local resolution map. b. Global resolution assessment by Fourier shell correlation at the 0.143 criterion.
