# Supplementary material for: UVC inactivation of pathogenic samples suitable for cryo-EM analysis
Source: Commun Biol. 2022 Jan 11;5:29. doi: 10.1038/s42003-021-02962-w (PMC8752862; doi:10.1038/s42003-021-02962-w)
Supplement: Supplementary file 2 — Reporting Summary [file 42003_2021_2962_MOESM2_ESM.pdf]

nature portfolio

Corresponding author(s): Ariane Briegel

Last updated by author(s): Nov 16, 2021

Reporting Summary

Nature Portfolio wishes to improve the reproducibility of the work that we publish. This form provides structure for consistency and transparency in reporting. For further information on Nature Portfolio policies, see our Editorial Policies and the Editorial Policy Checklist.

Statistics

For all statistical analyses, confirm that the following items are present in the figure legend, table legend, main text, or Methods section.

n/a	Confirmed	
☐	☒	The exact sample size (*n*) for each experimental group/condition, given as a discrete number and unit of measurement
☐	☒	A statement on whether measurements were taken from distinct samples or whether the same sample was measured repeatedly
☒	☐	The statistical test(s) used AND whether they are one- or two-sided<br>*Only common tests should be described solely by name; describe more complex techniques in the Methods section.*
☒	☐	A description of all covariates tested
☒	☐	A description of any assumptions or corrections, such as tests of normality and adjustment for multiple comparisons
☒	☐	A full description of the statistical parameters including central tendency (e.g. means) or other basic estimates (e.g. regression coefficient) AND variation (e.g. standard deviation) or associated estimates of uncertainty (e.g. confidence intervals)
☒	☐	For null hypothesis testing, the test statistic (e.g. *F*, *t*, *r*) with confidence intervals, effect sizes, degrees of freedom and *P* value noted<br>*Give P values as exact values whenever suitable.*
☒	☐	For Bayesian analysis, information on the choice of priors and Markov chain Monte Carlo settings
☒	☐	For hierarchical and complex designs, identification of the appropriate level for tests and full reporting of outcomes
☒	☐	Estimates of effect sizes (e.g. Cohen's *d*, Pearson's *r*), indicating how they were calculated

Our web collection on statistics for biologists contains articles on many of the points above.

Software and code

Policy information about availability of computer code

Data collection	cryo-EM data was collected using Thermo Fisher Scientific EPU v2.8.1 (SPA) or SerialEM v3.9.1 (tomography)
Data analysis	Data was analyzed using IMOD v4.11.1; Dynamo v9.6.0; ResMap v1.1.4; Relion 3.1.2; MotionCorr2; gCTF v1.06; CTFFIND-4.1.18; UCSF Chimera v1.13 or v1.14; ChimeraX; trRosetta; NAMD v2.13; VMD v1.9.4

For manuscripts utilizing custom algorithms or software that are central to the research but not yet described in published literature, software must be made available to editors and reviewers. We strongly encourage code deposition in a community repository (e.g. GitHub). See the Nature Portfolio guidelines for submitting code & software for further information.

Data

Policy information about availability of data

All manuscripts must include a data availability statement. This statement should provide the following information, where applicable:

- Accession codes, unique identifiers, or web links for publicly available datasets
- A description of any restrictions on data availability
- For clinical datasets or third party data, please ensure that the statement adheres to our policy

Structure related data for the ICP1 bacteriophage (UVC-treated and Control) capsid and the UVC-treated hApoF was deposited in the EMDB and PDB (ICP1 UVC-treated: EMD-13402, figure 2D-F ; ICP1 control: EMD-13403, figure 2C ; hApoF: EMDB-13364, PDB 7PF1 - figures 3, suppl figures 3 & 4, and suppl tables 1 & 2).

Field-specific reporting

Please select the one below that is the best fit for your research. If you are not sure, read the appropriate sections before making your selection.

- [x] Life sciences
- [ ] Behavioural & social sciences
- [ ] Ecological, evolutionary & environmental sciences

For a reference copy of the document with all sections, see nature.com/documents/nr-reporting-summary-flat.pdf

Life sciences study design

All studies must disclose on these points even when the disclosure is negative.

Sample size	For the cryo-ET structural study of the F6 chemotaxis array, we collected data on approximately 20 UVC-treated and 20 untreated cells. A subset of the data (cells containing the F6 array top view) were then used for particle picking and subtomogram averaging. For each sample, approximately 200 particles were used for the final average.<br>For the SPA ICP1 bacteriophage, 5282 and 12820 micrographs were collection from the control and UVC treated data, respectively. For the control treated ICP1, 25285 particles were autopicked and the final EM map was based on 4096 particles. For the UVC treated ICP1, 72048 particles were autopicked, of which 2845 particles were used for the final map. For the UVC-treated hApoF SPA, 1040 movies were collected resulting in 251,350 particles, of which 26,735 were used in the final average.
Data exclusions	For SPA. excluded particles were typically obstructed by other particles, lipid vesicles or other contaminants, or in some cases the carbon. For the STA, cells that did not contain a top view of the F6 chemotaxis array were excluded.
Replication	Experiments to confirm UVC inactivation of V. cholerae and ICP1 were carried out in triplicate and inactivation was confirmed by CFU and PFU, respectively. Subsequently, a separate set of vitrified samples were treated with UVC for a length of time resulting inactivation, one grid was used to confirm inactivation, the remaining grids were used for data collection by cryo-EM. For the SPA of hApoF, the sample was treated with UVC for the same time as the ICP1 sample, and one grid was imaged for data collection.
Randomization	For SPA, particles are randomly oriented, which is confirmed during analysis.
Blinding	N/A

Reporting for specific materials, systems and methods

We require information from authors about some types of materials, experimental systems and methods used in many studies. Here, indicate whether each material, system or method listed is relevant to your study. If you are not sure if a list item applies to your research, read the appropriate section before selecting a response.

Materials & experimental systems

n/a	Involved in the study	
[x]	[ ]	Antibodies
[x]	[ ]	Eukaryotic cell lines
[x]	[ ]	Palaeontology and archaeology
[x]	[ ]	Animals and other organisms
[x]	[ ]	Human research participants
[x]	[ ]	Clinical data
[x]	[ ]	Dual use research of concern

Methods

n/a	Involved in the study	
[x]	[ ]	ChIP-seq
[x]	[ ]	Flow cytometry
[x]	[ ]	MRI-based neuroimaging
